# Graph theoretic visualization of patient and health worker messaging in the EHR

**DOI:** 10.3389/frai.2024.1422208

**Published:** 2024-12-03

**Authors:** Muhammad Zia ul Haq, Andrew Hornback, Arash Harzand, David Andrew Gutman, Bradley Gallaher, Evan D. Schoenberg, Yuanda Zhu, May D. Wang, Blake Anderson

**Affiliations:** ^1^Noncommunicable Diseases and Mental Health Department, World Health Organization Regional Office for the Eastern Mediterranean, Cairo, Egypt; ^2^Bio-MIBLab, School of Computational Science and Engineering, Georgia Institute of Technology, Atlanta, GA, United States; ^3^Division of Cardiology, Emory University School of Medicine, Atlanta, GA, United States; ^4^Switchboard MD, Inc., Atlanta, GA, United States; ^5^Division of General Internal Medicine, Emory University School of Medicine, Atlanta, GA, United States

**Keywords:** artificial intelligence, data visualization, electronic health records, electronic medical records, graph visualization, network analysis

## Abstract

**Introduction:**

The electronic health record (EHR) has greatly expanded healthcare communication between patients and health workers. However, the volume and complexity of EHR messages have increased health workers' cognitive load, impeding effective care delivery and contributing to burnout.

**Methods:**

To understand these potential detriments resulting from EHR communication, we analyzed EHR messages sent between patients and health workers at Emory Healthcare, a large academic healthcare system in Atlanta, Georgia. We quantified the burden of messages interacted with by each health worker type and visualized the communication patterns using graph theory. Our analysis included 76,694 conversations comprising 144,369 messages sent between 47,460 patients and 3,749 health workers across 85 healthcare specialties.

**Results:**

On average, nurses/certified nursing assistants/medical assistants (nurses/CNA/MA) interacted with the most messages (350), followed by non-physician practitioners (NPP) (241), physicians (166), and support staff (155), with the average conversation involving 10.51 interactions before resolution. Network analysis of the communication flow revealed that each health worker was connected to approximately two other health workers (average degree = 2.10). In message sending, support staff led in closeness centrality (0.44), followed by nurses/CNA/MA (0.41), highlighting their key role in fast information spread. For message reception, nurses/CNA/MA (0.51) and support staff (0.41) also had the highest values, underscoring their vital role in the communication network on the receiving end as well.

**Discussion:**

Our analysis demonstrates the feasibility of applying graph theory to understand communication dynamics between patients and health workers and highlights the burden of EHR-based messaging.

## 1 Introduction

The electronic health record (EHR) contains rich and diverse data that offers significant insights into clinical practice (Zhang et al., 2016; Schrodt et al., 2020). While the adoption of EHR messaging has opened new channels for care coordination and improved patient-health worker relationships, there is evidence that rising message volumes have led to significant health worker burnout and even resulted in patient morbidity from delayed responses (Sittig and Singh, 2012; Casalino, 2017; Gardner et al., 2018; Jha et al., 2019; Chavez et al., 2020; De et al., 2021; Mermin-Bunnell et al., 2023).

In addition to the large volume of messages, inconsistent data harmonization and the complexity of interconnections in EHRs present challenges in retrieving and interpreting useful information (Yousefi et al., 2009; Chen et al., 2016; Zhang et al., 2016). Messages frequently have multiple recipients, which can exponentially increase the complexity of a conversation. As a result, currently available analysis and reporting tools are insufficient at communicating the underlying issues that may have the most significant impact on patient needs and outcomes (Lee and Hohler, 2014; Rashotte et al., 2016). Previous studies have targeted streamlining interfaces and enhancing usability to address cognitive burden (Shah et al., 2020). However, these initiatives have failed to reduce the intricate cognitive demands placed on health workers. Provider training programs have also been devised; however, they also show limited success in addressing the complex cognitive hurdles, intricate workflows, and health worker burnout associated with EHR communication (DiAngi et al., 2019). With regard to understanding healthcare communication, previous studies have primarily used social network analysis (SNA), often focusing on interpersonal interactions and information flow in clinical teams. For example, SNA has been used to understand referral patterns among physicians or collaboration networks in multidisciplinary teams (Sabot et al., 2017; Francis et al., 2024).

In contrast, our approach uses graph theory to study EHR communication networks, as network analysis of EHR communication could potentially help alleviate the complex relationships that previous methods have been unable to (Brunson and Laubenbacher, 2018; Moncho et al., 2021). Graph theory is a mathematical discipline focused on modeling the pairwise relationships between objects (Bollobás, 1998). Related data elements are represented as nodes (points or vertices in a graph) with edges (lines or connections between two nodes) (Bollobás, 1998; Schrodt et al., 2020). Recent studies have applied graph approaches in healthcare to study problems such as understanding trends in disease diagnoses, treatment, and clinical decision support (Soulakis et al., 2015; Zhang et al., 2016; Birtwell et al., 2019; Yang et al., 2019; Schrodt et al., 2020). For example, Soulakis et al. (2015) visualized EHR usage among healthcare providers treating heart failure patients, uncovering complex collaborative networks and multidisciplinary record access patterns, while Yang et al. (2019) proposed graphical modeling to optimize clinical information retrieval in EHRs.

This study aimed to investigate the communication dynamics in EHR-based patient-to-health worker and health worker-to-health worker conversations by identifying key participants, analyzing messaging patterns, and visually representing communication networks using graph theory. The specific objectives were to (1) classify health workers into distinct roles in the EHR system and quantify the communication burden borne by each health worker type; (2) visually represent the interactions between different health worker types and the associated message burden; and (3) extract key network parameters and statistics from the visualized communication networks.

## 2 Materials and methods

### 2.1 Study design and data sources

In this retrospective observational study, we analyzed messaging data from all outpatient clinics at Emory Healthcare, an integrated academic healthcare system in Atlanta, Georgia, USA. Deidentified metadata were extracted from PowerChart EHR (Oracle Cerner, Inc., Kansas City, MO) for all messages sent and received by health workers between March 1 and April 30, 2022. Our analysis mimicked the current workflows based on the desired destination of messages. The system was created using known workflows documented by the institution, and our analysis was designed to understand movement within the workflow protocol.

For a high-level understanding of communications, we classified health workers into four major categories: physicians, non-physician practitioners (NPP) (e.g., physician assistants and nurse practitioners), nurses/certified nursing assistants/medical assistants (nurses/CNA/MA), and support staff. A complete list of health worker titles for each category is provided in the Supplementary material. In this study, the term “health worker” refers to both clinical providers, such as physicians, NPPs, and nurses/CNA/MA, and non-clinical personnel, including administrative and operational support staff, all of whom play a role in healthcare communication and delivery. Communications within the dataset were categorized as touches, messages, and conversations. A message referred to any written communication between patients and health workers; a conversation was defined as a complete thread of messages; and a “touch” referred to each instance a health worker interacted with a message, such as reading, replying, or forwarding. The data were extracted in compliance with the Health Insurance Portability and Accountability Act (HIPAA) safe harbor provision. The study was deemed exempt from human subjects research by the Emory University Institutional Review Board and received a waiver of informed consent.

In preparation for data analysis, the raw messaging data were cleaned using Python 3.9. The data contained potential inconsistencies such as duplicate entries, missing values, and improperly formatted roles. We identified and removed duplicate messages based on unique message IDs to prevent skewing the analysis. Records with critical missing information (e.g., sender or receiver IDs) were excluded. A descriptive statistical analysis was performed to quantify the total communication burden on health workers by calculating metrics, including average conversations per health worker, average messages per health worker, average touches per conversation, and average touches per message. Burden of messages was calculated and plotted as the total number of messages interacted with by each health worker type. Furthermore, distinction was made between the proportion of messages where health workers were carbon copied (CC'ed) and those where they were intended to respond.

### 2.2 Graphical visualization

Graph theory (Schrodt et al., 2020) was applied to the messaging patterns between patients and healthcare workers using Gephi 0.10.1. Circular nodes represented message senders or receivers and edges represented the message paths. Each node was color-coded to represent distinct healthcare roles: physicians (blue), nurses/CNA/MA (pink), support staff (green), NPP (red), and patients (dark green).

The size of each node was proportional to the total number of messages the group interacted with, highlighting the communication burden on each role. Edges between nodes were directed, with arrows indicating the flow of messages from sender to receiver. The thickness of each edge corresponded to the frequency of messages exchanged between the groups. Edges were color-coded to enhance visual distinction, with specific colors representing different communication pathways (e.g., light blue arrow from physicians to patients, brown arrow from physicians to nurses/CNA/MA). Exact message counts were annotated on each edge to provide quantitative context. We applied the ForceAtlas2 layout algorithm to position nodes such that those with stronger connections were closer together. This layout emphasizes clusters within the network, revealing communication patterns and potential bottlenecks. Annotations and labels were added to highlight key communication pathways and differences between health worker types.

### 2.3 Communication network analysis

For a more comprehensive understanding of data distribution and the formation of clusters or groups (i.e., “communities”), we imported the complete parent dataset into Gephi (Gephi, 2024) after creating the relevant nodes and edges using Python 3.9. In this context, “communities” referred to clusters within the network where certain health workers (nodes) interacted more frequently or intensively among themselves than with other health workers in the network. To shed light on these communities and their characteristics, we applied the built-in Gephi algorithms to calculate average degree, network diameter, number of connected components, modularity, and average path length:

- **Average degree**–the average number of edges per node in the network.
- **Network diameter**–the longest shortest path between any two nodes in the network.
- **Number of connected components**–connected components are subgraphs directly or indirectly connected to each other but not to any nodes outside the subgraph in the network.
- **Modularity**–a measure of how well a network decomposes into modular communities. Ranging from 0 to 1, a low modularity score indicates a weak community structure, with uniform node distribution. High modularity scores indicate nodes are connected in dense clusters with fewer connections to outside clusters.
- **Average path length**–the average shortest distance between all pairs of nodes.

To understand the centrality of the network, we used three centrality measures for both senders and receivers:

- **Closeness centrality**–the relative importance of a node in a network, measured as the reciprocal of the sum of the lengths of the shortest paths of all other nodes to a node. Closeness centrality is measured on a scale from 0 to 1. See Equation 1 for the mathematical formula. In this equation,

- *C*(*v*) represents the closeness centrality of node *v*. It measures how close node *v* is to all other nodes in the network.
- *N* is the total number of nodes in the network.
- *d*(*u, v*) represents the shortest path distance between node *u* and node *v*. It represents the number of edges that need to be traversed to go from *u* to *v*.

- **Harmonic closeness centrality** - a modification of closeness centrality that reverses the sum and reciprocal operations and is set to 0 if there is no path between two nodes. Harmonic closeness centrality ranges from 0 to 1. See Equation 2 for the mathematical formula. In this equation,

- *H*(*v*) is the harmonic closeness centrality of node *v*. It's a variation of closeness centrality that handles disconnected graphs better.
- *d*(*u, v*) represents the shortest path distance between node *u* and node *v*.

- **Eigenvector closeness centrality**–used to measure influence of nodes in a network, assigning relative scores to nodes based on the concept of high-scoring nodes having connections that contribute more to the score. Thus, eigenvector closeness centrality is affected by the centrality of its neighbors as well as connections to other nodes. It ranges from 0 to 1. See Equation 3 for the mathematical formula. In this equation,

- *E*(*v*) is the eigenvector centrality of node *v*. It measures a node's influence in a network based on the influence of its neighbors.
- *x*_*v*_ is the eigenvector centrality score of node *v*.
- λ is the largest eigenvalue of the adjacency matrix of the graph. It normalizes the equation.
- *M*(*v*) is the set of nodes directly connected to the node *v*.
- *x*_*t*_ is the eigenvector centrality score of a neighboring node *t*.
- *a*_*v, t*_ is the entry in the adjacency matrix, representing the connection between node *v* and node *t*. It is 1 if node *v* and node *t* are connected and 0 otherwise.
- *G* is the entire set of nodes in the network.

We selected closeness centrality, harmonic closeness centrality, and eigenvector centrality as they are well-suited for analyzing communication efficiency and influence in structured healthcare environments. Other metrics in Gephi were not considered suitable for our specific analysis. For example PageRank, which measures node importance in a network, was not used because it assumes random navigation behavior and equates highly connected nodes with importance, which may be misleading in an EHR context where connectivity does not necessarily reflect the criticality of a role in patient care. Furthermore, we avoided metrics that depend on the size of a network, such as betweenness centrality, and focused only on metrics that are normalized between 0 and 1 and can thus be interpreted in the same manner if the analysis were applied to a different communication network that may differ greatly in size than the one we analyzed.

### 2.4 Oversight

Switchboard MD (Atlanta, GA) provided funding for the study. The study was designed by investigators at Emory University and the Georgia Institute of Technology with input from the sponsor. Primary data extraction was performed by the senior author (BA) who is employed by the sponsor. The data were analyzed by the first and second author (MZH and A. Hornback). The sponsor's remaining affiliate authors (DAG, BG, EDS and YZ) did not have access to the study data and participated in data interpretation and revision of the manuscript only. Additional details of the individual author contributions are included at the end of the manuscript.

## 3 Results

The dataset included a total of 144,369 messages in 76,694 conversations sent between 47,460 patients and 3,749 health workers, across 85 different specialties, as shown in Table 1. On average, each health worker participated in 33.01 conversations (SD 48.93) and 44.51 messages (SD 66.26). Each conversation had an average of 10.51 touches (SD 22.76) while each message had an average of 5.58 touches (SD 8.80). Given the large standard deviations, we analyzed the median and interquartile range values to understand how outliers were shifting the data. As shown in Table 1, the median values for each of the four metrics were lower than the average, reflecting the effect of a larger burden being placed on a portion of health workers. For instance, the median number of conversations per health worker was 14.00, with an interquartile range of 41.00, while the median number of messages per health worker was 18.00, with an interquartile range of 52.00.

**Table 1:** Descriptive statistics of communication variables.

**Communication variable**	**Value**
Total patients	47,460
Total health workers	3,749
Total specialties	85
Total conversations	76,694
Total messages	144,369
Average conversations per health worker (SD)	33.01 (48.93)
Median conversations per health worker (IQR)	14.00 (41.00)
Average messages per health worker (SD)	44.51 (66.26)
Median messages per health worker (IQR)	18.00 (52.00)
Average touches per conversation (SD)	10.51 (22.76)
Median touches per conversation (IQR)	5.00 (7.00)
Average touches per message (SD)	5.58 (8.80)
Median touches per message (IQR)	3.00 (3.00)

**Figure 1:**
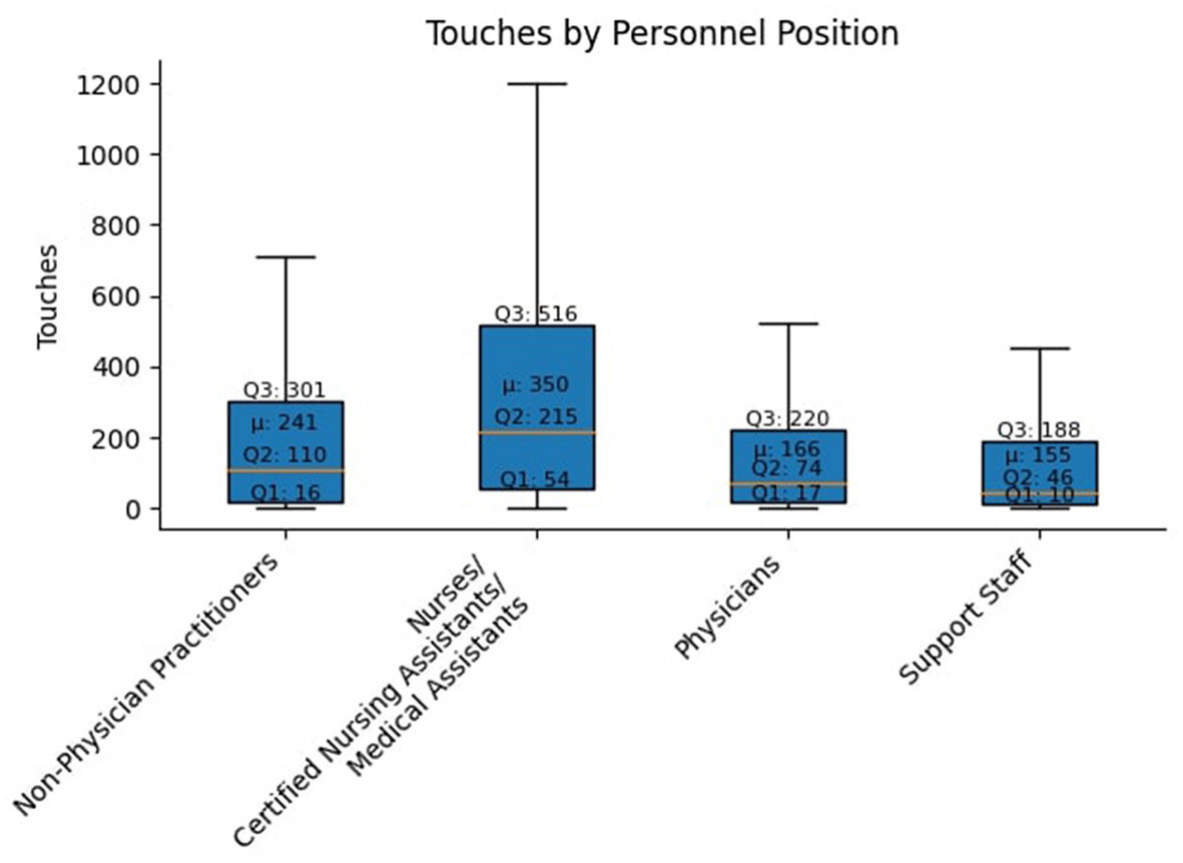
The median values for each health worker classification were below the mean, reflecting large outliers by various health workers in each classification with a much heavier message touch burden.

**Figure 2:**
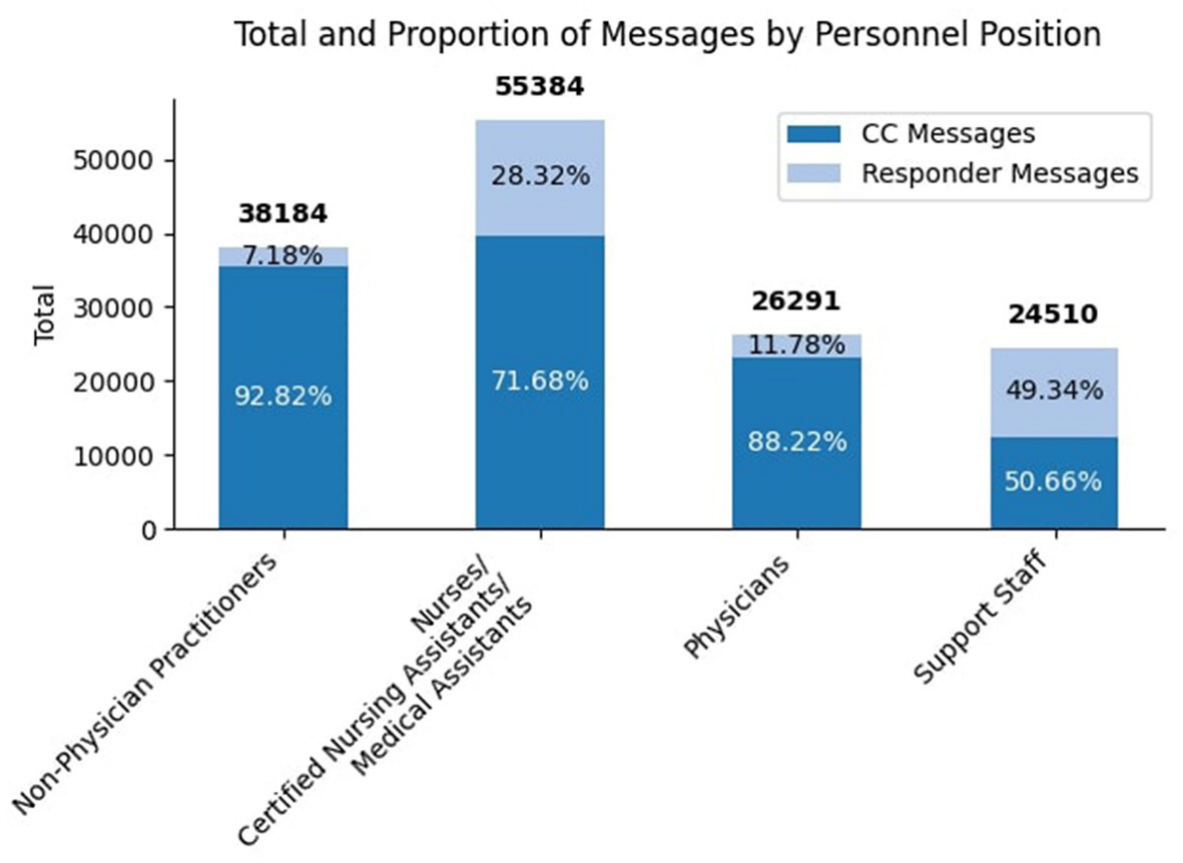
Distribution of EHR messages among healthcare roles. The bar chart quantitatively depicts the total counts of CC'ed and responded messages managed by nurses/CNA/MA, NPP, physicians, and support staff. Each bar is proportionally segmented to reflect the relative contribution of CC'ed and responded messages, with the total number of messages for each role displayed above.

Figure 1 visualizes the message touch burden by health worker classification. Nurses/CNA/MA on average touched the most messages, with an average slightly above 350, compared to the median of 215 for this group. Similar patterns were seen in other health worker classifications. A more detailed analysis of message types (CC'ed messages vs. responded messages) across roles is presented in Figure 2. Nurses/CNA/MA managed the highest overall volume, with 55,384 messages, of which 71.68% were CC'ed messages (39,700), and 28.32% were responded messages (15,684), indicating actionable items. NPPs had a total of 38,184 messages, with 92.82% being CC'ed messages (35,446) while 7.18% were responded messages (2,738). Similarly, physicians handled 26,291 messages, of which 88.22% were CC'ed messages (23,193), leaving 11.78% as responded messages (3,098). Interestingly, support staff exhibited a more balanced communication profile. Out of their 24,510 messages, 50.66% were CC'ed messages (12,413) and 49.34% were responded messages (12,097).

### 3.1 Graphical visualization

**Figure 3:**
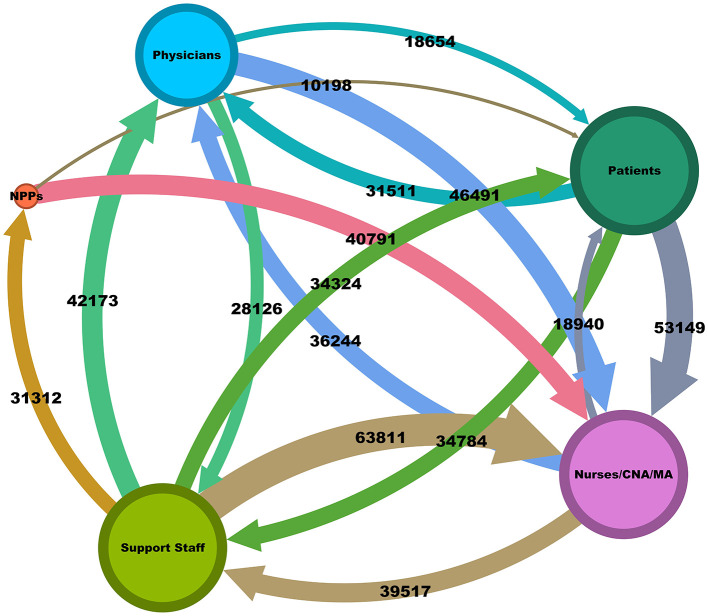
A visualization of the conversation network in EHR showing stops and message flow. Each node, representing a health worker type, has a size proportional to the number of messages it interacts with, while the arrows and edge width denoting the direction and frequency of message flow, respectively. Thicker edges indicate higher message frequencies, quantified by exact numbers for clarity. Bidirectional message exchanges are depicted, showcasing the dynamic interplay of communication between health workers.

The Gephi graph visualization in Figure 3 provides a comprehensive view of the EHR message network, highlighting the roles of various health workers in communication dynamics. Support staff played a central role, managing the largest number of message exchanges across different groups. For instance, they sent 63,811 messages to nurses/CNA/MA and 42,173 messages to physicians, reinforcing their function as a central conduit of communication within the healthcare system. Nurses/CNA/MA also exchanged a large volume of messages with physicians (36,244 messages sent) and support staff (39,517 messages sent). This pattern underscored the pivotal role of nurses/CNA/MA in bridging communication between clinical and support teams.

Patient communication was largely directed toward nurses/CNA/MA (53,149 messages) and support staff (34,784 messages). In contrast, physicians received fewer direct messages from patients (31,511), aligning with their more specialized focus on clinical decision-making rather than direct patient interaction. NPPs had a lower total message volume but still played a key role in communication, particularly with nurses/CNA/MA, sending 40,791 messages.

This comprehensive view reflected the findings related to overall touches depicted in Figure 1 and the message types in Figure 2, confirming that nurses/CNA/MA and support staff bear the heaviest communication burdens. In both views, nurses/CNA/MA have a larger volume of message activity than all other health workers. Related to Figure 2, it is implicit that a larger portion of the number of messages sent to nurses/CNA/MA require a response (they are not CC messages) compared to other health workers as well. It can also be deduced that the high number of overall touches shown in Figure 1 for nurses/CNA/MA and the absolute measures shown in Figure 3 can explain a significant portion of the difference in the average messages per health worker of 44.51 and the median or 18.00 shown in Table 1 as being attributable to just one category of health workers.

### 3.2 Communication network analysis

The analysis of network metrics for message routing among different health worker types, as detailed in Table 2, revealed an average degree of 2.10. This degree indicated that each participant was typically connected to two others, suggesting a balanced and moderately dense communication network that facilitates collaboration without overwhelming individual participants.

**Table 2:** Overview of Communication Network Characteristics.

**Network statistic**	**Value**
Average degree	2.10
Network diameter	2.00
Number of connected components	1.00
Modularity	0.40
Average path length	1.00^a^
Number of nodes	144,378
Numbers of edges	303,269

^a^The average path length is displayed as 1.00 for precision purposes. The true value for the average path length was 1.00006, as an average path length of 1.00 would indicate each node of the graph was connected, and the network diameter would also have to equal 1.00. Here, the network diameter of 2.00 indicates that some individuals were not connected via an edge (message) in the network, but that the vast majority were, hence the slightly greater than 1.00 exact average path length.

The network's efficiency was underscored by a network diameter of 2.00 and an average path length of 1.00 (1.00006, precisely, as an average path length of 1.00 would indicate all nodes are connected by an edge, and thus, the network diameter would also have to equal 1.00.) Here, the average path length and network diameter indicated that the vast majority of individuals are connected on at least one message, with a small population not being directly connected, but only having one intermediary, leading to a network diameter of 2.00). The network modularity of 0.40 indicated somewhat defined communities within the network, which could represent departments or specialty groups that tend to communicate amongst themselves almost as often as other departments or specialty groups.

As shown in Table 3, for senders within the network, support staff and nurses/CNA/MA exhibited the highest closeness centrality scores of 0.44 and 0.41, respectively, indicating their central roles in the communication network. Harmonic closeness centrality was also highest for support staff and nurses/CNA/MA at 0.57 and 0.52, respectively. The same pattern held for eigenvector centrality as well, with 0.51 for nurses/CNA/MA and 0.68 for support staff. In terms of message reception, nurses/CNA/MA showed the highest closeness centrality, harmonic closeness centrality, and eigenvector centrality at 0.51, 0.68, and 1.00, respectively, indicating their proximity and accessibility to other network participants. Similarly, support staff showed relatively high scores as receivers, indicating their importance and prevalence in the communication network.

**Table 3:** Communication Dynamics Sender Analysis.

**Health worker Role**	**Receiver or sender**	**Closeness**	**Harmonic closeness**	**Eigenvector closeness**
NPP	Receiver	0.35	0.38	0.14
NPP	Sender	0.34	0.36	0.07
Nurses/CNA/MA	Receiver	0.51	0.68	1.00
Nurses/CNA/MA	Sender	0.41	0.52	0.51
Physicians	Receiver	0.39	0.48	0.42
Physicians	Sender	0.36	0.41	0.22
Support Staff	Receiver	0.41	0.51	0.51
Support Staff	Sender	0.44	0.57	0.68

Notably, physicians as senders achieved a harmonic closeness centrality of 0.41, mirroring the closeness centrality score of nurses, indicating their essential role in initiating communication within the network. NPP had relatively consistent values for all three measures as senders and receivers, with harmonic closeness centrality being the largest in both mediums at 0.36 as senders and 0.38 as receivers. These statistics, though lower than those for support staff, nurses and clinic support staff, highlight their critical role in the information flow.

## 4 Discussion

This study of EHR based messaging networks between patients and providers visually depicts the complexity of interactions within a large academic healthcare system. Our results highlight that nurses/CNA/MA and support staff serve as the primary conduits of communication, handling the highest volume of messages and acting as critical intermediaries between patients, physicians, and NPP. The predominance of CC'ed messages in the communication patterns of nurses/CNA/MA and NPP suggests that much of their workload is related to being copied on information, with only a fraction of messages requiring direct action. This dynamic, while essential for ensuring information flow, may also contribute to cognitive overload and operational inefficiencies, particularly for roles that are already under significant pressure. The small network diameter and average path length, reflect that the EHR messaging system allows for quick dissemination of information with only a few intermediaries needed, which is beneficial for timely communication. The clear centrality of nurses/CNA/MA and support staff, as evidenced by high closeness and eigenvector centrality scores, underscores their important role in EHR communication. It also raises concerns regarding potential bottlenecks, where too much information is relayed through a few particular health workers. It is important to note, that physicians and NPP also shoulder a significant communication burden. In the context of healthcare delivery, the communication load on nurses/CNA/MA and physicians is critical, as they are primarily involved in critical clinical care.

Our findings agree with previous literature indicating that the rise in data quantity in the EHR system is overwhelming and can contribute to health worker burnout (Yousefi et al., 2009; Zhang et al., 2016; Emanuel et al., 2020). Our research adds to this discourse by providing a quantitative and visual representation of communication dynamics, highlighting the significant roles and burdens borne by different health worker types. The heavy burden on nurses/CNA/MA is consistent with prior research that links EHR usage with increased workloads and potential burnout, particularly for nursing staff, Kutney-Lee et al. (2021) highlighting that the insights gained from our analysis are not confined to Emory Healthcare. The identified communication patterns and bottlenecks are likely reflective of broader trends in healthcare communication, especially in large, integrated health systems.

A major strength of this study is the novel application of graph theory to EHR communication data, offering a detailed visual and quantitative representation of message flow across healthcare roles. While previous studies have used SNA to understand healthcare communication (Sabot et al., 2017; Francis et al., 2024), our approach differs by leveraging graph theory specifically to model EHR-based messaging networks. It provides a more granular and data-driven visualization of communication dynamics. Unlike traditional SNA, which may rely on surveys or observational data, our method utilizes actual messaging records, enhancing the objectivity and scale of the analysis. This methodology provides actionable insights that could inform future efforts to optimize EHR systems, streamline workflows, and reduce communication inefficiencies. Additionally, the large dataset enhances the robustness of our findings, allowing for generalizable insights into communication patterns in a major healthcare system.

However, the study has limitations that should be considered when interpreting the results. Most notably,the data were derived from a single EHR system, Cerner Now Oracle Health at Emory Healthcare. The fundamental principles of graph theory are universal and can be applied to any system where communication data can be extracted and structured similarly. However, institutional workflows and policies may vary at different healthcare facilities and EHR systems. Therefore, when applying our method to other settings, it is crucial to account for these variations to ensure accurate interpretation of the communication networks. Without a clear understanding of the specific processes that govern message routing and task delegation, it is difficult to determine whether the observed communication burdens are the result of inefficiencies or inherent job responsibilities.

Furthermore, the study did not account for interactions outside the EHR system, such as face-to-face communication or phone calls, which could provide a more holistic understanding of health worker-patient interactions. The omission of these interactions means the communication network presented is incomplete, potentially underestimating the true connectivity and workload of health workers. Including multi-modal communication data including phone calls, and surveys in future studies would provide a more comprehensive understanding of health worker communication dynamics and workload distribution.

One of the most unexpected findings was the relatively low number of responded messages handled by NPP, despite their important clinical role. This may suggest that NPP primarily serve as recipients of information rather than actively engaging in message responses, potentially due to task delegation within care teams. This observation contrasts with their expected clinical responsibilities and raises questions about the distribution of communication tasks within healthcare teams. Additionally, the nearly equal distribution of CC'ed and responded messages among support staff highlights their dual role in administrative and clinical coordination, a finding that may warrant further investigation into whether this balance is optimal or if it places unnecessary burdens on these health workers.

The identification of nurses/CNA/MA and support staff as central nodes in the communication network suggests that they are potential bottlenecks in message flow. To alleviate their burden and improve communication efficiency, healthcare organizations can consider implementing automated triage systems using artificial intelligence and machine learning algorithms. These systems can categorize and prioritize messages based on urgency and content, directing them to the appropriate health worker with minimal delay. Additionally, redistributing certain administrative tasks from overburdened roles to underutilized staff can balance the workload and reduce burnout risk.

Optimizing EHR workflows by customizing notification settings and reducing unnecessary CCs can also minimize cognitive overload. Training programs focusing on effective communication practices within the EHR may enhance team coordination and ensure that messages requiring action are promptly addressed. Future studies should investigate the direct impact of the EHR messaging burden on health worker burnout and patient outcomes, as well as explore potential solutions.

## 5 Conclusion

This study at Emory Healthcare represents the utility of graph theory to examine EHR communication networks, uncovering critical insights into the roles of nurses/CNA/MA and support staff as central communicators in the system. The findings highlight the complexity of health worker interactions, with significant communication loads being unevenly distributed across health worker roles. The study underscores the need for targeted redesign of EHR systems to better manage communication burdens, streamline workflows, and reduce the risk of cognitive overload on health workers.


(1) $$\begin{array}{l} C\left(v\right)=\frac{N-1}{\sum_{u}d\left(u,v\right)} \end{array}$$

(2) $$\begin{array}{l} H\left(v\right)=\underset{u|u\neq v}{\sum }\frac{1}{d\left(u,v\right)} \end{array}$$

(3) $$\begin{array}{l} E\left(v\right)=x_{v}=\frac{1}{\lambda }\underset{t\in M\left(v\right)}{\sum }x_{t}=\frac{1}{\lambda }\underset{t\in G}{\sum }a_{v,t}x_{t} \end{array}$$

## Data Availability

The datasets presented in this article are not readily available because the participants of this study did not give written consent for their data to be shared publicly, so due to the sensitive nature of the research supporting data is not available. Requests to access the datasets should be directed to blake@switchboardmd.com.
